# Air‐side ammonia stripping coupled to anaerobic digestion indirectly impacts anaerobic microbiome

**DOI:** 10.1111/1751-7915.13482

**Published:** 2019-09-18

**Authors:** Nuria Fernandez‐Gonzalez, Chiara Pedizzi, Juan M. Lema, Marta Carballa

**Affiliations:** ^1^ Department of Chemical Engineering Universidade de Santiago de Compostela Rúa Lope Gómez de Marzoa, s/n. 15782 Santiago de Compostela Spain; ^2^ Present address: Present address: Department of Chemical Engineering and Environmental Technology Valladolid University Dr. Mergelina, s/n 47011 Valladolid Spain

## Abstract

Air‐side stripping without a prior solid–liquid phase separation step is a feasible and promising process to control ammonia concentration in thermophilic digesters. During the process, part of the anaerobic biomass is exposed to high temperature, high pH and aerobic conditions. However, there are no studies assessing the effects of those harsh conditions on the microbial communities of thermophilic digesters. To fill this knowledge gap, the microbiomes of two thermophilic digesters (55°C), fed with a mixture of pig manure and nitrogen‐rich co‐substrates, were investigated under different organic loading rates (OLR: 1.1–5.2 g COD l^−1 ^day^−1^), ammonia concentrations (0.2–1.5 g free ammonia nitrogen l^−1^) and stripping frequencies (3–5 times per week). The bacterial communities were dominated by *Firmicutes* and *Bacteroidetes* phyla, while the predominant methanogens were *Methanosarcina* sp archaea. Increasing co‐substrate fraction, OLR and free ammonia nitrogen (FAN) favoured the presence of genera *Ruminiclostridium*,* Clostridium* and *Tepidimicrobium* and of hydrogenotrophic methanogens, mainly *Methanoculleus* archaea. The data indicated that the use of air‐side stripping did not adversely affect thermophilic microbial communities, but indirectly modulated them by controlling FAN concentrations in the digester. These results demonstrate the viability at microbial community level of air side‐stream stripping process as an adequate technology for the ammonia control during anaerobic co‐digestion of nitrogen‐rich substrates.

## Introduction

Anaerobic digestion (AD) is a microbial process based on the combined and syntrophic activities of a wide range of microorganisms from hydrolytic and fermentative bacteria to methanogenic archaea (Stams and Plugge, 2009). The microbial community of AD reactors is mainly affected by substrate composition (Regueiro *et al*., 2012; Zhang *et al*., 2014) and operational conditions (Vanwonterghem *et al*., 2015; Regueiro *et al*., 2016; Amha *et al*., 2018). One of the most important factors that drastically affects AD microbial communities is temperature as it imposes a selection pressure on the communities shifting the abundance and activity of specific populations in digesters treating different kinds of waste like beet molasses, maize, slaughterhouse or fish residues, municipal solid wastes and swine or pig manures (Regueiro *et al*., 2014; Vrieze *et al*., 2015; Lin *et al*., 2016). Thermophilic (45–60°C) communities are regarded as less diverse and even than mesophilic (30–45°C) ones. Under high temperatures, microbial communities are usually dominated by *Firmicutes* phylum and hydrogenotrophic methanogens, whereas *Bacteroidetes* and acetoclastic archaea are the prevalent groups under mesophilic conditions in reactors treating sewage sludge or co‐digesting food waste and slaughterhouse residues (Sundberg *et al*., 2013). In these thermophilic communities, syntrophic oxidizing bacteria and hydrogenotrophic methanogens play a significant role. Particularly, hydrogen‐producing bacteria from the genus *Clostridium*, together with syntrophic bacteria of the genera *Pelotomaculum, Syntrophomonas* and *Coprothermobacter*, associated with *Methanoculleus*,* Methanosarcina* or *Methanobrevibacter* hydrogenotrophic methanogens have been observed in anaerobic reactors fed with biowaste and sewage sludge (Ritari *et al*., 2012), food waste (food waste; Guo *et al*., 2014), cattle manure (Moset *et al*., 2015) and maize silage (Pap *et al*., 2015).

The organic loading rate (OLR) is another important factor shaping AD microbial communities. Organic overloading causes process imbalance, and thus volatile fatty acid (VFA) accumulation, and a shift in the microbial community (Guo *et al*., 2014; Regueiro *et al*., 2015). Overloading events are also associated with an increase in free ammonia nitrogen (FAN) concentrations in the digesters when the substrate used is rich in nitrogen compounds such as manure (De Francisci *et al*., 2015; Regueiro *et al*., 2016; Sun *et al*., 2016) and some types of food waste (Gao *et al*., 2015; Yun *et al*., 2016). High levels of ammonia can inhibit microbial activities (Liu and Sung, 2002; Zhang *et al*., 2012; Westerholm *et al*., 2018) although it has been demonstrated that acclimatization of microbial communities can alleviate this inhibition (Yan *et al*., 2019). It is well known that acetoclastic methanogens are less tolerant to ammonium than hydrogenotrophic archaea (Angelidaki and Ahring, 1993; Vrieze *et al*., 2012; Westerholm *et al*., 2012, 2018; Yan *et al*., 2019), and consequently, increasing ammonium concentration shifts methanogenesis from acetoclastic to hydrogenotrophic pathway (Lü *et al*., 2013; Werner *et al*., 2014; Regueiro *et al*., 2016). In contrast, less is known about the effects of high ammonium concentrations on bacterial organisms. Recent studies have reported the dominance of *Bacteroidetes* group under low‐ammonium conditions, while under high concentrations the phylum *Cloacimonetes* disappears and the *Firmicutes* phylum, in particular *Clostridiales* and *Lactobacillales* orders, dominates the community (Werner *et al*., 2014; Vrieze *et al*., 2015; Regueiro *et al*., 2016; Westerholm *et al*., 2018). When high ammonia levels inhibit obligate acetoclastic methanogens, the microbial community shifts towards syntrophic acetate oxidation pathway (Westerholm *et al*., 2016) although the keystone taxa responsible for the function are unclear (Werner *et al*., 2014; Theuerl *et al*., 2019).

Some strategies have been developed to avoid ammonia AD inhibition (Krakat *et al*., 2017). Among them, side‐stream ammonia stripping has modest reagent costs and easy operation and allows the recovery of nitrogen as ammonium sulfate (Zhang *et al*., 2012; Pedizzi *et al*., 2017). During this process, the digester content (digestate) is treated in a separate column where it is exposed to increased pH and temperature causing the transfer of dissolved ammonia to gas phase which is removed through the application of a gas flow, usually air. Then, the stripped digestate is recirculated back into the reactor (Bousek *et al*., 2016; Pedizzi *et al*., 2017). When the stripping process lacks a previous solid–liquid separation step, the complete digestate from the reactor enters the stripping column, and therefore, a fraction of the reactor microbial community is exposed to the harsh conditions of the stripping process. It is well established that the conditions applied during the stripping process (high temperature, high pH and the presence of oxygen at saturation) alter AD microbial communities. High‐pH conditions inhibit methanogenic archaea (Yuan *et al*., 2006; Chen *et al*., 2017), and high oxygen concentrations modify microbial composition by removing *Clostridium* senso stricto 1, which negatively impacts the capacity for hydrogen production (Yang and Wang, 2018). Finally, high temperature correlates with lower microbial community diversity and complexity (Karakashev *et al*., 2005; Westerholm *et al*., 2018). But, to the best of our knowledge, the combination of high temperature, pH and oxygen levels on AD microbiome has not been assessed previously. The aim of this work was to study the effects of applying an air side‐stream stripping column without a previous solid–liquid separation step on the thermophilic microbial community of anaerobic digesters under different ammonia concentrations, stripping frequencies and organic loading rates.

## Results and discussion

### Reactor performance during microbiome analysis

**Figure 1:**
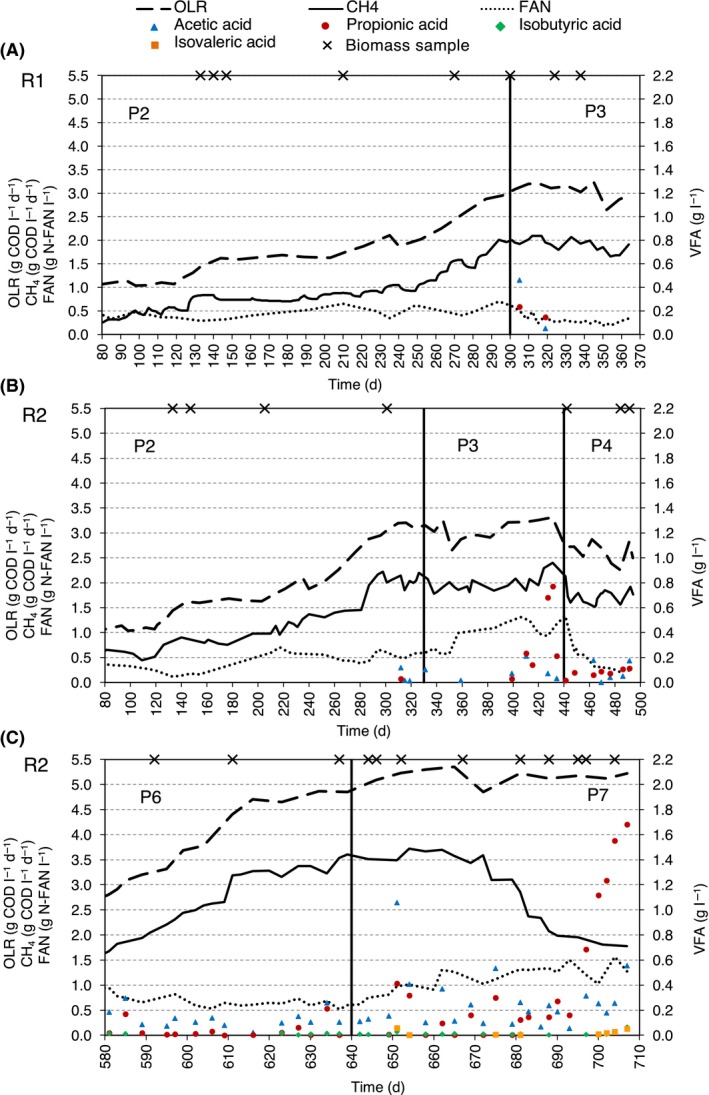
Organic loading rate (OLR), methane production, free ammonia nitrogen (FAN) and main volatile fatty acid (VFA) concentrations in the reactors during the experiment. (A) Periods 2 and 3 of R1; (B) Periods 2, 3 and 4 of R2; and (C) Periods 6 and 7 of R2. Vertical lines separate operational periods. Crosses indicate biomass sampling days.

The operation of the two reactors (R1 and R2) throughout the periods during which anaerobic microbiome was monitored is shown in Fig. 1. A detailed description of reactor operation and of the stripping unit can be found in Pedizzi *et al*. (2017). In brief, after the initial start‐up and acclimation to thermophilic conditions (Period 1, from day 0 to day 79), co‐digestion was started in both reactors with the addition of maize silage and Ecofrit^®^ (Period 2: 80–299 days in R1 and 80–329 in R2). The OLR was increased from 1.0 to 3.0 g chemical oxygen demand (COD) l^−1^ day^−1^, whereas FAN concentration rose from 0.2 to 0.6 g N‐FAN l^−1^ (Fig. 1A and B). In Period 3 of R1 (300–365 days), the stripping process was applied by treating and recirculating 21% of the reactor volume three times per week. This lowered FAN concentration from 0.6 to 0.2 g N‐FAN l^−1^, while both OLR and methane production remained stable (Fig. 1A). In R2, Period 3 (330–439 days) corresponded to the sensible increase of FAN concentration from 0.6 to 1.3 g N‐FAN l^−1^ throughout a time span of almost 100 days, while the OLR was kept constant, leading to a lack of negative effects on methane production (Fig. 1B). During Period 4 of R2 (440–494 days), the stripping process was applied under the same conditions as in Period 3 of R1, but with a frequency of five times per week, decreasing FAN levels from 1.3 to 0.2 g N‐FAN l^−1^, whereas OLR lowered slightly to 2.5–2.8 g COD l^−1^ day^−1^ (Fig. 1B) and methane production remained stable. After a maintenance interval of 2 months (Period 5 of R2), in Period 6 of R2 (580–639 days) the stripping process was used again three times per week to maintain FAN concentration close to 0.6 g N‐FAN l^−1^ while the OLR was increased from 2.5 to 5.0 g COD l^−1^ day^−1^ by incrementing the co‐substrate fraction in the feeding (Fig. 1C). Consequently, methane production rose from 1.6 to 3.0 g COD l^−1^ day^−1^ resulting in a COD removal efficiency of 65%. During Period 7 of R2 (640–707 days), side‐stream stripping was stopped and FAN rose sharply from 0.6 to 1.5 g N‐FAN l^−1^ (OLR remained at 5.1 g COD l^−1^ day^−1^), causing VFA accumulation and leading to final digestion collapse (Fig. 1C).

### Overview of community diversity

**Figure 2:**
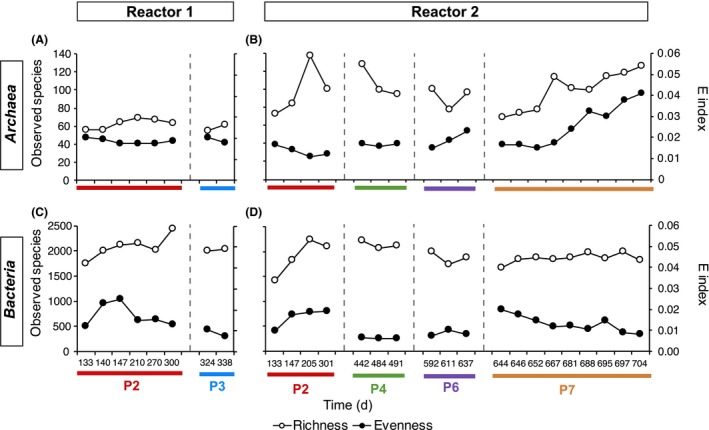
Temporal changes in archaeal and bacterial estimated richness and evenness for R1 (A, C) and R2 (B, D). The different operational periods are indicated in the horizontal axis. The Simpson *E* index ranges from 0 to 1, where 1 implies total community uniformity.

A total of 787 053 bacterial and 1 295 823 archaeal high‐quality 16S rRNA gene partial sequences were obtained for the 28 biomass samples (Table S1). Community richness (number of species) and evenness (equality of abundance of species) estimations showed a less diverse and complex archaeal community compared to bacterial microbiome (Fig. 2), in concordance with previous observations of biogas‐producing communities (Weiss *et al*., 2008; Wirth *et al*., 2012). Bacterial richness and evenness tended to increase during P2, the phase in which the OLR and FAN increased from 1.0 to 3.0 g COD l^−1^ day^−1^ and from 0.2 to 0.6 g N‐FAN L^−1^ respectively (Fig. 1), especially in R2. Later, while richness become constant, bacterial evenness oscillated with time to notably decrease in P7, the phase in which R1 was operated at high OLR (5.1 g COD l^−1^ day^−1^) but stripping was not applied with the consequent FAN increment to 1.5 g N‐FAN l^−1^ and VFA accumulation (Fig. 1). Archaeal richness and evenness were constant to some extent in R1, whereas in R2, they fluctuated with time, including a remarkably increasing trend in P7 in both measurements. The trends observed in R2 during P7 seem to be related to the FAN levels in the reactor (Fig. 1C). Lower levels of bacterial uniformity related to high ammonia levels in AD have been previously observed (Werner *et al*., 2014) and might be related to ammonia inhibition of many species of bacteria. By contrast, the increase in archaeal community richness and evenness with FAN levels is probably related to the promotion of new species of methanogens that are probably incoming with the feeding and that can face the high‐FAN conditions.

### Linkage of community dynamics and operational conditions

**Figure 3:**
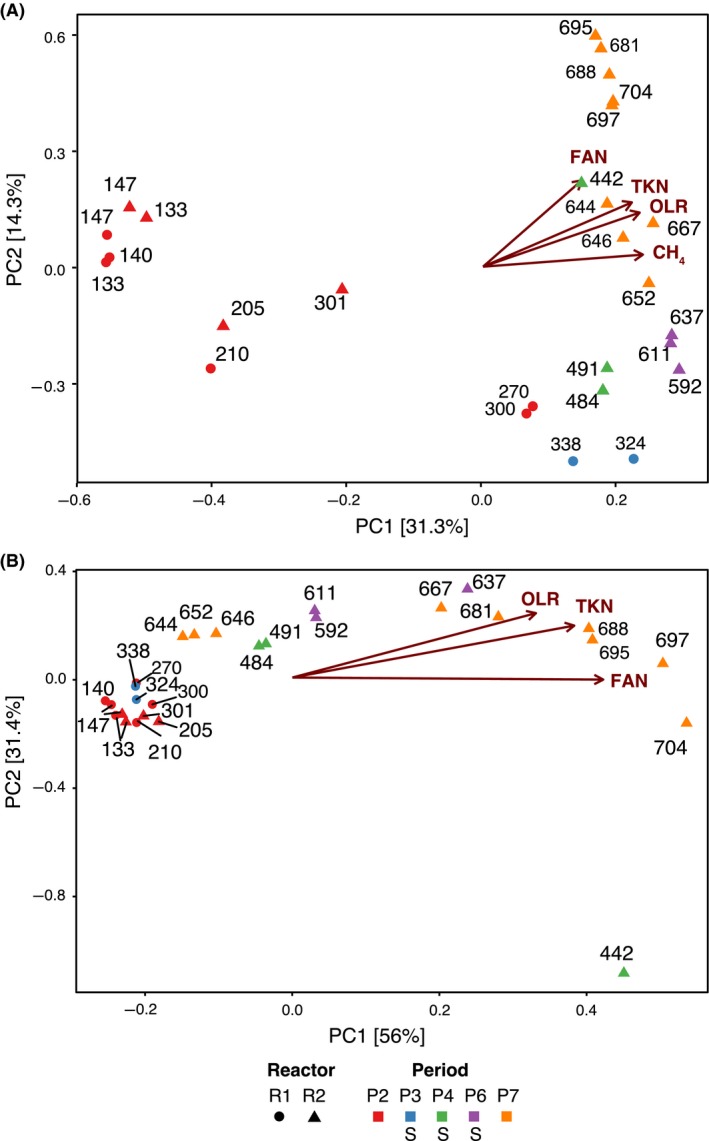
tb‐PCA showing community structure changes and its correlation with operational parameters for bacteria (A) and archaea (B). Each point represents the community composition of a reactor (indicated by the form shape) at a given time point (sample labels indicate operational day; form colour indicates the period). Vectors indicate the increasing values of operational variables (only the statistically significant variables (*P*‐value < 0.05) are shown). Periods in which the biomass was exposed to stripping are indicated with an S in the legend.

Overall, bacterial and archaeal community structure was dynamic through time and the changes were mainly correlated with alterations in the OLR and FAN levels (Fig. 3, S2–S5). In P2 of both reactors, bacterial but not archaeal community structure shifted in parallel to a moderate OLR increase from 1 to 3 g COD l^−1^ day^−1^, which also caused FAN to rise from 0.2 to 0.6 g N‐FAN l^−1^ due to the introduction of co‐substrates.

To study the decoupled effects of FAN and OLR, FAN was gradually increased from 0.6 to 1.3 g N‐FAN l^−1^ in R2 between P2 and P4 (days 330–439; Fig. 1) while keeping a constant OLR (Pedizzi *et al*., 2017). In this case, bacterial and archaeal community structures were different (Fig. 3) before (day 301) and after the FAN increase (day 442). During P4 of R2, when the stripping unit was used to decrease the FAN levels from 1.3 to 0.2 g N‐FAN l^−1^, while the OLR was still constant, both archaeal and bacterial community structures largely changed again (Fig. 3). These data indicate the strong influence that FAN concentrations impose to microbial communities. Then, the effect of increasing OLR was tested by keeping a low and constant FAN by applying the stripping process during P6 (Fig. 1). In this case, the overall community structure was not very altered, apart from archaeal communities that become more dissimilar when the OLR reached values close to 5 g COD l^−1^ day^−1^ (day 637; Fig. 3B). In P7, a high OLR (5.1 g COD l^−1^ day^−1^) was maintained without the application of the stripping process, resulting in a raise of FAN concentration from 0.6 to 1.5 g N‐FAN l^−1^ and increasing concentrations of VFAs (Fig. 1). In this case, the structures of both communities were gradually modified (Fig. 3). These results confirm previous observations on the importance of OLR and FAN levels on community organization of AD reactors under both mesophilic (Werner *et al*., 2014; Regueiro *et al*., 2015, 2016) and thermophilic conditions (Yenigün and Demirel, 2013; Kuroda *et al*., 2015). Interestingly, the largest community shift of this period occurred earlier in archaeal than in bacterial communities. The largest *Archaea* community modification occurred between days 652 and 667 when the increasing FAN concentrations crossed the boundary of 1 g N‐FAN l^−1^, when a peak in the concentrations of VFAs was observed and when methane production started a stepwise decrease. However, the largest bacterial community change occurred later, between days 667 and 688. The latter does not agree with Li *et al*. (2017), who observed that bacterial communities responded earlier during mesophilic solid‐state AD of protein‐rich organic wastes.

During P3, P4 and P6 when stripping process was applied, part of the biomass was temporarily exposed to high pH, temperatures and oxygen concentrations, conditions that individually can impair AD communities (Karakashev *et al*., 2005; Yuan *et al*., 2006; Yang and Wang, 2018). However, neither bacterial nor archaeal community structure data clustered by the application of stripping (Fig. 3; S1). These observations were confirmed by the nonparametric multivariate analysis of variance (PERMANOVA) (Anderson, 2001) that was applied to test the influence of stripping and FAN on community composition. One critical assumption of a PERMANOVA is that the variance between the groups of samples compared needs to be similar, or the PERMANOVA can produce in a false‐positive result. In the case of the bacterial communities, the variance homogeneity assumption was not met for the stripping condition (Pr: 0.002). In that case, the PERMANOVA was not reliable as it could lead to a false‐positive result; therefore, the analysis was not performed. In the case of archaeal communities, the variance was homogeneous among the conditions considered: stripping (Pr > 0.7) and FAN (Pr > 0.9). Accordingly one way PERMANOVA analysis indicated the significant separation of bacterial community according to FAN levels (F: 5.4564, Pr: 0.001). For archaeal communities, two‐way PERMANOVA indicated that while they did not differ according to stripping (F: 2.052, Pr > 0.1), communities exposed to different FAN concentrations were different (F: 40.799, Pr: 0.001). The results suggest that the effects of stripping process on thermophilic AD microbial communities are negligible compared with the strong pressures imposed by FAN concentrations and other operational factors.

### Taxonomic composition of bacterial community

*Bacteria* community was mainly composed of *Firmicutes* organisms, which on average represented 80.2 ± 7.6% of *Bacteria*, followed by *Bacteroidetes* (8.6 ± 5.5%) and *Thermotogae* (6.1 ± 5.7%) (Fig. S6A). Dominance of *Firmicutes* and *Bacteroidetes* phyla and the presence of *Thermotogae* phylum are common under thermophilic AD conditions (Ritari *et al*., 2012; Werner *et al*., 2014).

**Figure 4:**
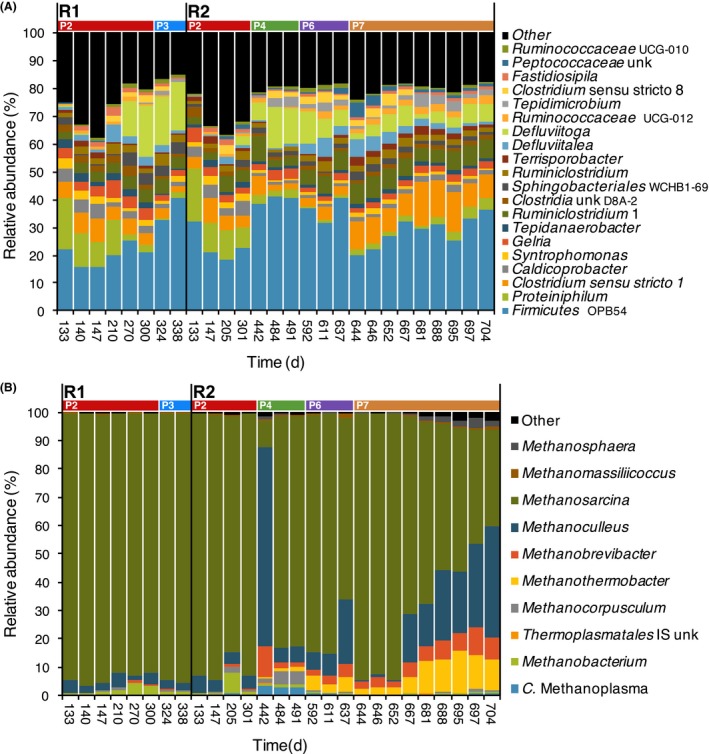
Microbial community composition of AD digesters. A. Relative abundances of the 20 top most abundant bacteria genera, which individually represented over the 1.5% of the bacterial community in at least one sample. B. Ten top most abundant archaea genera, which individually represented over the 0.4% of the archaeal community in at least one sample. Operational periods are indicated on top with coloured horizontal bars. IS, incertae sedis; unk, unknown.

The bacteria of the *Firmicutes* phylum were very diverse, although two taxa represented together 70% of the bacteria (Table S2): the *Clostridiales* order (41.1 ± 9.3%) and the uncultured *Firmicutes* OPB54 group (29.0 ± 8.2%). A high presence of *Clostridiales* order is also characteristic of AD reactors (Moset *et al*., 2015; Cardinali‐Rezende *et al*., 2016). However, the taxonomy and metabolic capacities of the unknown group *Firmicutes* OPB54, which was the most abundant bacterial genus through the entire experiment (Fig. 4A), are still unclear as only one species has been isolated so far (Liu *et al*., 2014), and most of the knowledge about the group is limited to environmental clones found in a wide range of habitats including anaerobic reactors (Dunfield *et al*., 2012). The other *Firmicutes* organisms were mostly strict or facultative anaerobic bacteria with broad fermentative capabilities (Niu *et al*., 2009), like the genera *Clostridium, Defluvitalea* and *Tepidimicrobium* (Fig. 4A), or more specialized fermentative genus like *Ruminiclostridium,* whose members degrade complex carbohydrates such as cellulose, xylan and cellobiose (Yutin and Galperin, 2013), and *Caldicoprobacter* bacteria*,* which degrade hemicelluloses and mono‐ and oligosaccharides (Yokoyama *et al*., 2017). Many of these taxonomic groups, such as *Ruminiclostridium*,* Defluvitalea* or *Caldicoprobacter,* contain thermophilic organisms. Among *Bacteroidetes*, the most abundant genus was *Proteiniphilum*, which mostly hydrolyses and ferments proteins (Sakamoto, 2014), and within the *Thermotogae* phylum, *Defluviitoga* genus, with a single described species that ferments carbohydrates to acetate, H_2_ and CO_2_ (Hania *et al*., 2012).

Interestingly, the diversity of syntrophic bacteria in the studied reactors was large. It included organisms of the *Syntrophomonadaceae* family and *Thermoanaerobacterales* order like *Gelria* ssp. (Table S2), a group of thermophilic, saccharolytic and fermentative bacteria (Plugge *et al*., 2002). Moreover, some other bacterial taxa that have been either confirmed or suggested to belong to the syntrophic acetate‐oxidizing (SAO) bacteria were detected as well, like *Tepidanaerobacter* (Westerholm *et al*., 2011) and *Syntrophaceticus* (Westerholm *et al*., 2010). Other proposed, although not yet confirmed, SAO bacteria present in R1 and R2 were *Coprothermobacter,* a proteolytic genus commonly found in manure‐based digesters (Tsapekos *et al*., 2017), C*lostridium* sp. and *Thermotogae* organisms (Westerholm *et al*., 2016).

### Taxonomic composition of archaeal community

Most archaea belonged to the *Euryarchaeota* phylum (99.98 ± 0.04%; Table S3), mainly from the *Methanomicrobia* (91.3 ± 7.9%) and *Methanobacteria* (7.7 ± 7.6%) classes (Fig. S6B). *Methanosarcina* genus dominated archaeal communities for most of the operational period (Fig. 4B). *Methanosarcina* is an acetoclastic methanogen that also uses the hydrogenotrophic pathway. It is a robust and adaptable methanogen that grows at different temperature ranges and tolerates abrupt changes on it, a capacity probably related to the presence of heat stress‐ and cold stress‐regulating genes in its genome (Vrieze *et al*., 2012). For that reason, it is not surprising that *Methanosarcina* members are often reported as the main acetoclastic methanogens at elevated total ammonia, salt or volatile fatty acid concentrations and dominate the methanogenic communities in thermophilic digesters fed with manure (Karakashev *et al*., 2005; Vavilin *et al*., 2008; Vrieze *et al*., 2012, 2015; Moset *et al*., 2015). Other hydrogenotrophic methanogens, mostly from the genera *Methanoculleus*,* Methanothermobacter* and *Methanobrevibacter*, also represented an important fraction of the archaeal community (Fig. 4), especially during day 442 and P7 of R2, periods in which FAN levels were high (Fig. 1). *Methanoculleus* *,* which can dominate pig manure reactors (Barret *et al*., 2013), comprises robust methanogens that thrive under thermophilic or ammonia stress (Pap *et al*., 2015; Poirier *et al*., 2016), and its members are often reported as partners of SAO bacteria (Westerholm *et al*., 2016). The rise of *Methanoculleus* genus during P7 of R2 occurred in parallel to the drop in methane production (Figs 1 and 4), which could lead to consider this genus as an early indicator of AD process failure. However, there are examples of well‐functioning AD reactors under high ammonia concentrations with communities dominated by this genus (Pap *et al*., 2015; Poirier *et al*., 2016).

### Dominance and dynamics of co‐occurring groups of microorganisms

**Figure 5:**
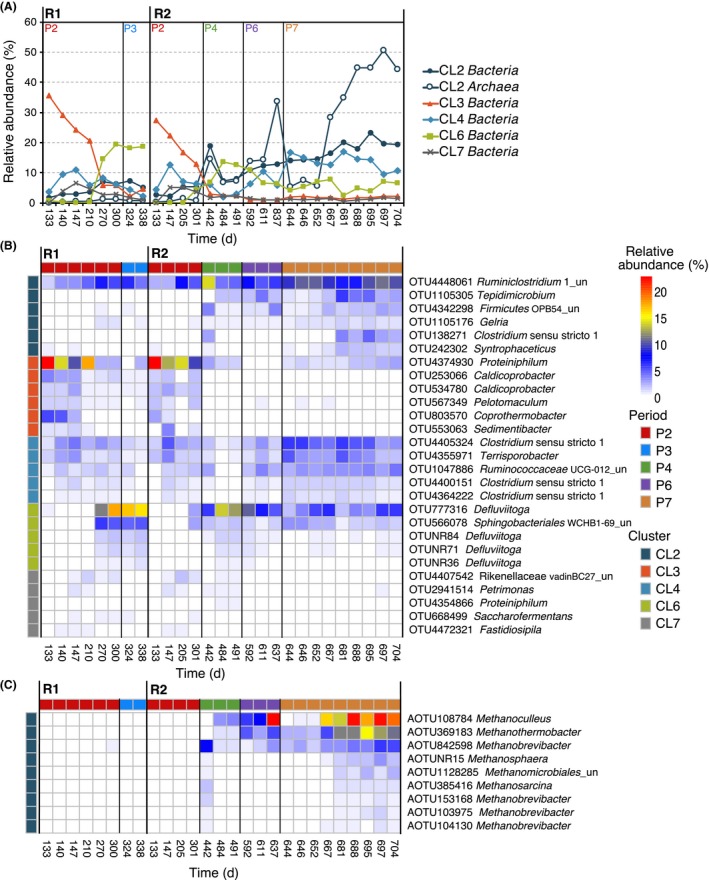
Temporal trends of main clusters of co‐occurring microorganisms and their most relatively abundant OTUs. A: Accumulated relative abundance of OTUs in the clusters by domain. Heatmaps of the most abundant OTUs within each cluster (relative abundances over 0.25% in at least one observation) for bacteria (B) and archaea (C). Coloured cells on the left indicate the cluster, and cells on the top designate the periods. Only data from CL2, CL3, CL4, CL6 and CL7 are shown.

To find potential associations among microorganisms, a co‐occurring network was calculated for the OTUs with relative abundances higher than 0.1%. Twenty‐five groups of co‐occurring OTUs called clusters (CL) were found (Figs S7 and S8), each of them showing a unique temporal trend and taxonomic profile (Figs 5A and S8). The clusters that contained more than 10 OTUs (CL2, CL3, CL4, CL6 and CL7) were selected for further analysis. The two largest clusters, CL2 and CL3, seemed to be antagonist because they showed opposite temporal trends and relationships with OLR, FAN and VFA concentrations (Figs 5A, S9). CL3 dropped during Period 2, when the OLR was moderately increased by the addition of the co‐substrates. Indeed, relative abundance of CL3 was negatively correlated with increasing levels of OLR, FAN and VFAs (Fig. S9). Most microorganisms in CL3 are specialized in the degradation of proteinaceous substrates: mostly the proteolytic bacteria of the genus *Proteiniphilum* (on average, 40.8% of bacteria within the cluster; Fig. 5B), accompanied by syntrophic bacteria such as those from the proteolytic genus *Coprothermobacter* (7.7% of CL3), consisting of acetate‐degraders in syntrophic association with hydrogenotrophic methanogens (Ho *et al*., 2014), and *Pelotomaculum* (4.9% of CL3), a genus of syntrophic propionate‐oxidizing bacteria (Bok *et al*., 2005). Changes in microbial communities linked to transitions from mono‐ to co‐digestion have been previously observed (Fitamo *et al*., 2017) and can be expected given the strong influence of substrates on AD microbial communities (Regueiro *et al*., 2012).

In contrast, the relative abundances of CL2 microorganisms increased under high levels of OLR and FAN, especially during P6 and P7 (Fig. 5). Indeed, CL2 was positively correlated with OLR, FAN and VFAs (Fig. S9). CL2 was dominated by *Ruminiclostridium* bacteria (42% of bacteria within CL2), which hydrolyse complex carbohydrates. Although high ammonium content inhibits the glycolysis pathway during AD, thus impeding carbohydrate degradation (Mata‐Alvarez *et al*., 2000), the microbial consortia of these reactors seem to become adapted. Cardinali‐Rezende *et al*. (2016) already found carbohydrate‐degrading communities adapted to high ammonium concentrations in AD. Syntrophic bacteria composed a significant fraction of CL2, although in this case, the SAO *Tepidimicrobium* (10% of bacteria within CL2) and *Gelria* (7.2%) bacteria were the most abundant. CL2 also contained a diverse group of hydrogenotrophic methanogens (*Methanoculleus* (43% of *Archaea* within the cluster), *Methanothermobacter* (24%) and *Methanobrevibacter* (20%); Fig. 5C).

CL6 was mainly formed by *Defluviitoga* OTUs (79.3% of cluster microorganisms), which reaches its highest abundance under moderate OLR levels (P2, P3, P4; Fig. 5B). Bacteria of both *Defluviitoga* and *Ruminiclostridium* genera hydrolyse complex carbohydrates, and it is plausible that these organisms compete at moderate organic loads but *Ruminiclostridium* likely displaces *Defluviitoga* when OLR and FAN increase.

The other two most abundant clusters (CL4 and CL7) mainly contained organisms that use a wider range of substrates. CL4 was composed of OTUs, such as *Clostridium* (51.9% of CL4) or *Terrisporobacter* sp. (15.7% of CL4) species that degrade a broad array of simple substrates like amino acids or mono‐ and disaccharides. Most microorganisms in CL4 are likely to be involved in the acidogenesis step of the AD, since they are present throughout the experiment, although they tend to become more abundant during periods with high OLR (Fig. 5B, S9) and less abundant in P7, indicating a lower overall resistance to high FAN and VFA concentrations. Likewise, CL7 contained OTUs with diverse metabolic capabilities, although their abundances are quite low (Fig. 6B), for instance *Petrimonas* (17.8% of the cluster), whose members degrade carbohydrates and some organic acids, or *Fastidiosipila* (16.0%), which is a genus of proteolytic bacteria (Falsen *et al*., 2005). CL7 is probably a group of transient microorganisms with almost no correlation with any operational parameter (Fig. S9).

To conclude, this work shows that even though part of the thermophilic biomass was periodically exposed to the stripping conditions (65°C, high pH and oxygen at saturation levels), overall community structure was not predominantly affected by those harsh conditions. Instead, the thermophilic communities were mainly shaped by the organic loading rate, FAN and VFA concentrations, variables that are interrelated. Hence, air‐side stripping indirectly modulated microbial communities by controlling FAN concentrations in the digester allowing to optimize the treatment of N‐rich wastes.

## Experimental procedures

### Experimental set‐up and sampling

Two (R1 and R2) continuous stirred tank reactors (CSTR) with 14L working volume were operated in thermophilic range (55 ± 1°C) during 365 and 707 days respectively. The feeding consisted of a mixture of pig manure, maize silage and Ecofrit^®^, and the experiment was divided into seven operating periods. During Period 3 (P3) of R1 and Periods 4 (P4) and 6 (P6) of R2, the reactors were connected to a stripping column, which worked at 65°C for 3 h with an airflow of 1 L_air_ L_digestate_^−1^ min^−1^ without pH control in batch mode, treating 21% of the reactor volume in each batch. The stripping column was operated three times per week in P3 (R1) and P6 (R2) and five times per week in P4 (R2) to investigate the effects of higher recirculating ratio. A detailed description of the operation of reactors and the stripping column can be found in Pedizzi *et al*. (2017). Five 1‐ml aliquots of well‐homogenized biomass samples were taken on days 133, 140, 147, 210, 270, 300, 324 and 338 of R1 operation and days 133, 147, 205, 301, 442, 484, 491, 592, 611, 637, 644, 646, 652, 667, 681, 688, 695, 697 and 704 for R2 and immediately frozen at −20°C until further analysis. During the periods when the stripping was applied, biomass samples were always taken before feeding and 30 min after recirculating the stripped digestate into the anaerobic reactor.

### DNA extraction and 16S rRNA gene amplicon sequencing

DNA was extracted using the PowerSoil DNA Isolation Kit (MoBio Laboratories, Inc., Carlsbad, NM, USA) following the manufacturer's instructions. DNA concentrations were quantified by fluorometry (Qubit fluorometer; Thermo Fisher Scientific, Waltham, MA, USA), and standard electrophoresis was used to assess DNA size and integrity. Amplicon libraries were prepared for fragments of the 16S rRNA gene of both bacteria and archaea domains with primers including Illumina adaptors and barcodes. The V3V4 region of the bacterial 16S rRNA gene was amplified with the primer pair S‐D‐Bact‐0341‐b‐S‐17 and S‐D‐Bact‐0785‐a‐A (Klindworth *et al*., 2013). In the case of *Archaea*, the V2V3 region was amplified with the primer set Arch1F and Arch1R as previously described (Cruaud *et al*., 2014). Prepared DNA libraries were analysed for quality in a Bioanalyzer (Bioanalyzer; Agilent Technologies, Santa Clara, CA, USA) and subsequently quantified by qPCR. The multiplexed libraries were pooled in equimolar amounts and sequenced at the genomics unit of the Parque Científico de Madrid (Spain) on an Illumina MiSeq System (Illumina, San Diego, CA, USA) using MiSeq Reagent Kit v3 (Illumina).

### Sequence processing

The paired‐end sequence reads were demultiplexed and trimmed to remove Illumina adapters, barcodes and primers. After truncating read length to 250 bp to remove low‐quality ends, paired reads were merged as previously described (Eren *et al*., 2013), discarding any sequence with quality scores below 30 and any indetermination. The obtained high‐quality sequences were clustered into operational taxonomic units (OTUs) at a 97% cut‐off for 16S rRNA gene identity with the open‐reference OTU‐picking method using UCLUST (Edgar, 2010) and SILVA v123 database (Quast *et al*., 2013) in QIIME v.1.9.1 (Caporaso *et al*., 2010b; Rideout *et al*., 2014). Representative sequences from each OTU were aligned using PyNAST (Caporaso *et al*., 2010a). Any unaligned sequence, singletons, OTUs classified as unknown or to the incorrect domain and chimeric sequences analysed with VSEARCH (Rognes *et al*., 2016) were removed from further analysis. Raw reads are available at the Short Read Archive (SRA) of the NCBI under the accession number SRP133112.

### Data analysis

To calculate diversity estimations data was rarefied to 20 000 and 20 700 sequences for archaea and bacteria data sets respectively. Richness was determined as the estimated number of species, and evenness was measured with the Simpson evenness (*E* index):

$$E\;\text{index}=\frac{1}{\sum_{i=1}^{S}p_{i}^{2}}\times \frac{1}{S},$$

where *S* is the number of observed species (richness) and *p*_*i*_ is the proportion of species *i* relative to the total number of species. *E* takes values from 0 to 1, with 1 being complete evenness or uniformity. Beta diversity, the degree of community differentiation between samples, was measured with Bray–Curtis dissimilarities using Hellinger‐transformed OTU data (Legendre and Gallagher, 2001) and visualized using transformation‐based principal component analysis (tb‐PCA). Additionally, Bray–Curtis dissimilarities were analysed by average linkage clustering. PERMANOVAs were performed using Bray–Curtis dissimilarity matrices. First, the analysis of multivariate homogeneity of group dispersions was performed using the Anderson procedure (Anderson, 2006) through the betadisper and anova functions for each factor tested. Next, PERMANOVA was performed with the adonis function including 999 permutations. Co‐occurring microbial networks were constructed with CoNet v.1.1.1.beta (Faust *et al*., 2012) in Cytoscape v.3.4.0 (Shannon *et al*., 2003). The input data matrix included only OTUs with relative abundances over 0.1% (522 OTUs). Compositional effects were avoided using the bootstrap‐renormalization procedure (Faust *et al*., 2012). The co‐occurrence measures employed were as follows: Spearman and Pearson correlations, Bray–Curtis and Kullback–Leibler dissimilarities and their *P*‐values merged. The Benjamini–Hochberg procedure was used to correct for multiple comparisons, discarding edges that were not significant (*q* ≤ 0.05). Only edges supported by at least two measurements were kept. The resulting network was then analysed to detect highly interacting groups of nodes (clusters) using the GLay algorithm (Su *et al*., 2010). Pearson's correlations were calculated between total relative abundance of clusters and OLR, FAN, CH_4_ production and VFA concentrations. All statistical analyses were performed in R (R Core Team, 2016) using the R packages vegan (Oksanen *et al*., 2018), phyloseq (McMurdie and Holmes, 2013) and Rhea (Lagkouvardos *et al*., 2017).

## Conflicts of interest

None declred.

## Supporting information


**Fig. S1**. Clustering of Bray Curtis dissimilarities of bacteria (A) and archaea (B) communities. Samples exposed to stripping are in red and bold.
**Fig. S2**. tb‐PCA of archaeal community structure changes and their correlations with operational parameters for R1 (A) and R2 (B). Each point represents the community composition of a reactor at a given time point (sample labels indicate operational day**;** form color indicates the period). Vectors indicate the increasing values of operational variables (only the statistically significant variables (*P*‐value < 0.05) are shown.
**Fig. S3**. tb‐PCA of bacterial community structure changes and their correlations with operational parameters for R1 (A) and R2 (B). Each point represents the community composition of a reactor at a given time point (sample labels indicate operational day**;** form color indicates the period). Vectors indicate the increasing values of operational variables (only the statistically significant variables (*P*‐value < 0.05) are shown.
**Fig. S4.** tb‐PCA showing archaeal community structure changes and N‐FAN concentrations. Each point represents the community composition of a reactor (indicated by the form shape) at a given time point (sample labels indicate operational day). The color scale indicates the N‐FAN concentrations on each time point.
**Fig. S5.** tb‐PCA showing bacterial community structure changes and N‐FAN concentrations. Each point represents the community composition of a reactor (indicated by the form shape) at a given time point (sample labels indicate operational day). The color scale indicates the N‐FAN concentrations on each time point.
**Fig. S6.** The relative abundances of the most abundant taxa are shown for: A: 10 top bacterial phyla and B: 3 top archaeal classes. Operational periods are indicated on top with colored horizontal bars.
**Fig. S7**. Co‐occurring network of the abundant OTUs (relative abundance > 0.1%). Each node represents an individual OTU that is linked with other OTUs with similar temporal patters. Highly interconnected nodes have been separated into 25 clusters. Cluster number is indicated on top. For major clusters the number of OTUs within the cluster is shown in parenthesis. Node color indicates the phylum of the OTU.
**Fig. S8.** Normalized relative abundances of OTUs belonging to the clusters with more than 10 OTUs (CL2, CL3, CL4, CL6 and CL7). Each row shows the OTUs within one of the clusters. Left column correspond to values from R1 and right column to values from R2.
**Fig. S9.** Pearson correlations between bacterial relative abundances of clusters and volatile fatty acids, free ammonia nitrogen (FAN, g NH_3_‐N l^−1^), organic loading rate (OLR, g COD l^−1^ day^−1^) and methane production (g COD l^−1^ day^−1^). Color corresponds to Pearson´s r values (blue for positive and red for negative). Circle diameter indicates the *P*‐value.

**Table S1.** Number of high quality sequences obtained per sample. Sample name indicates: reactor, period and sampling day.

**Table S2**. Relative abundances of the 10 more abundant Bacteria classes, orders and families. Values are in percentages of total Bacteria.

**Table S3**. Relative abundances of the *Archaea* phylum and 10 more orders and families. Values are in percentages of total *Archaea*.
