# Supplementary material for: Air‐side ammonia stripping coupled to anaerobic digestion indirectly impacts anaerobic microbiome
Source: Microb Biotechnol. 2019 Sep 18;12(6):1403–16. doi: 10.1111/1751-7915.13482 (PMC6801131; doi:10.1111/1751-7915.13482)
Supplement: Supplementary file 1 — Fig. S1. Clustering of Bray Curtis dissimilarities of bacteria (A) and archaea (B) communities. Samples exposed to stripping are in red and bold. Fig. S2. tb‐PCA of archaeal community structure changes and their correlations with operational parameters for R1 (A) and R2 (B). Each point represents the community composition of a reactor at a given time point (sample labels indicate operational day; form color indicates the period). Vectors indicate the increasing values of operational variables (only the statistically significant variables (P‐value < 0.05) are shown. Fig. S3. tb‐PCA of bacterial community structure changes and their correlations with operational parameters for R1 (A) and R2 (B). Each point represents the community composition of a reactor at a given time point (sample labels indicate operational day; form color indicates the period). Vectors indicate the increasing values of operational variables (only the statistically significant variables (P‐value < 0.05) are shown. Fig. S4. tb‐PCA showing archaeal community structure changes and N‐FAN concentrations. Each point represents the community composition of a reactor (indicated by the form shape) at a given time point (sample labels indicate operational day). The color scale indicates the N‐FAN concentrations on each time point. Fig. S5. tb‐PCA showing bacterial community structure changes and N‐FAN concentrations. Each point represents the community composition of a reactor (indicated by the form shape) at a given time point (sample labels indicate operational day). The color scale indicates the N‐FAN concentrations on each time point. Fig. S6. The relative abundances of the most abundant taxa are shown for: A: 10 top bacterial phyla and B: 3 top archaeal classes. Operational periods are indicated on top with colored horizontal bars. Fig. S7. Co‐occurring network of the abundant OTUs (relative abundance > 0.1%). Each node represents an individual OTU that is linked with other OTUs with similar temporal [file MBT2-12-1403-s001.docx]

**Air-side ammonia stripping coupled to anaerobic digestion indirectly impacts anaerobic microbiome**

**SUPPLEMENTARY DATA: FIGURES**

**Nuria Fernandez-Gonzalez, Chiara Pedizzi, Juan M. Lema and Marta Carballa**

Department of Chemical Engineering, Institute of Technology, Universidade de Santiago de Compostela, 15782 Santiago de Compostela, Galicia, Spain

* Corresponding autor: nuria.fernandez.gonzalez@uva.es

**A**

**B**

**Figure S1**. Clustering of Bray Curtis dissimilarities of bacteria (A) and archaea (B) communities. Samples exposed to stripping are in red and bold.

**A**

**B**

**Figure S2**. tb-PCA of archaeal community structure changes and their correlations with operational parameters for R1 (A) and R2 (B). Each point represents the community composition of a reactor at a given time point (sample labels indicate operational day**;** form color indicates the period). Vectors indicate the increasing values of operational variables (only the statistically significant variables (p-value < 0.05) are shown.

**A**

**B**

**Figure S3**. tb-PCA of bacterial community structure changes and their correlations with operational parameters for R1 (A) and R2 (B). Each point represents the community composition of a reactor at a given time point (sample labels indicate operational day**;** form color indicates the period). Vectors indicate the increasing values of operational variables (only the statistically significant variables (p-value < 0.05) are shown.

**Figure S4.** tb-PCA showing archaeal community structure changes and N-FAN concentrations. Each point represents the community composition of a reactor (indicated by the form shape) at a given time point (sample labels indicate operational day). The color scale indicates the N-FAN concentrations on each time point.

**Figure S5.** tb-PCA showing bacterial community structure changes and N-FAN concentrations. Each point represents the community composition of a reactor (indicated by the form shape) at a given time point (sample labels indicate operational day). The color scale indicates the N-FAN concentrations on each time point.

**Figure S6.** The relative abundances of the most abundant taxa are shown for: A: 10 top bacterial phyla and B: 3 top archaeal classes. Operational periods are indicated on top with colored horizontal bars.

**Figure S7**. Co-occurring network of the abundant OTUs (relative abundance > 0.1%). Each node represents an individual OTU that is linked with other OTUs with similar temporal patters. Highly interconnected nodes have been separated into 25 clusters. Cluster number is indicated on top. For major clusters the number of OTUs within the cluster is shown in parenthesis. Node color indicates the phylum of the OTU.

**Figure S8.** Normalized relative abundances of OTUs belonging to the clusters with more than 10 OTUs (CL2, CL3, CL4, CL6 and CL7). Each row shows the OTUs within one of the clusters. Left column correspond to values from R1 and right column to values from R2.

**Figure S9.** Pearson correlations between bacterial relative abundances of clusters and volatile fatty acids, free ammonia nitrogen (FAN, g NH_3_-N L^-1^), organic loading rate (OLR, g COD L^-1^ d^-1^) and methane production (g COD L^-1^ d^-1^). Color corresponds to Pearson´s r values (blue for positive and red for negative). Circle diameter indicates the *p*-value.
