# Supplementary material for: Air‐side ammonia stripping coupled to anaerobic digestion indirectly impacts anaerobic microbiome
Source: Microb Biotechnol. 2019 Sep 18;12(6):1403–16. doi: 10.1111/1751-7915.13482 (PMC6801131; doi:10.1111/1751-7915.13482)
Supplement: Supplementary file 2 — Table S1. Number of high quality sequences obtained per sample. Sample name indicates: reactor, period and sampling day. [file MBT2-12-1403-s002.pdf]

Table S1. Number of high quality sequences obtained per sample. Sample name indicates: reactor, period and sampling day.

Sample	*Bacteria*	*Archaea*
R1.P2.133	31490	34553
R1.P2.140	24396	80557
R1.P2.147	21946	64055
R1.P2.210	33180	86818
R1.P2.270	24081	100194
R1.P2.300	27393	67909
R1.P3.324	31718	66602
R1.P3.338	25552	71550
R2.P2.133	33881	63065
R2.P2.147	24780	49264
R2.P2.205	20788	38201
R2.P2.301	27964	45931
R2.P4.442	25281	20092
R2.P4.484	27671	37294
R2.P4.491	31963	39220
R2.P6.592	28165	40310
R2.P6.611	30716	44003
R2.P6.637	30678	26475
R2.P7.644	27033	67864
R2.P7.646	33502	45524
R2.P7.652	34075	43405
R2.P7.667	24623	27924
R2.P7.681	35086	33278
R2.P7.688	29631	24636
R2.P7.695	31680	28439
R2.P7.697	36301	24034
R2.P7.704	33479	24626
Average	29150	47993
Total	787053	1295823
